# Artificial intelligence for telemedicine diabetic retinopathy screening: a review

**DOI:** 10.1080/07853890.2023.2258149

**Published:** 2023-09-21

**Authors:** Luis Filipe Nakayama, Lucas Zago Ribeiro, Frederico Novaes, Isabele Ayumi Miyawaki, Andresa Emy Miyawaki, Juliana Angélica Estevão de Oliveira, Talita Oliveira, Fernando Korn Malerbi, Caio Vinicius Saito Regatieri, Leo Anthony Celi, Paolo S. Silva

**Affiliations:** a Institute for Medical Engineering and Science, Massachusetts Institute of Technology, Cambridge, MA, USA; b Department of Ophthalmology, São Paulo Federal University, Sao Paulo, Brazil; c Federal University of Parana, Curitiba, Brazil; d Pontifical Catholic University of Parana, Curitiba, Brazil; e Department of Biostatistics, Harvard TH Chan School of Public Health, Boston, MA, USA; f Department of Medicine, Beth Israel Deaconess Medical Center, Boston, MA, USA; g Beetham Eye Institute, Joslin Diabetes Centre, Harvard Medical School, Boston, MA, USA; h Philippine Eye Research Institute, University of the Philippines, Manila, Philippines

**Keywords:** Telemedicine, artificial intelligence, diabetic retinopathy, fairness

## Abstract

**Purpose:**

This study aims to compare artificial intelligence (AI) systems applied in diabetic retinopathy (DR) teleophthalmology screening, currently deployed systems, fairness initiatives and the challenges for implementation.

**Methods:**

The review included articles retrieved from PubMed/Medline/EMBASE literature search strategy regarding telemedicine, DR and AI. The screening criteria included human articles in English, Portuguese or Spanish and related to telemedicine and AI for DR screening. The author’s affiliations and the study’s population income group were classified according to the World Bank Country and Lending Groups.

**Results:**

The literature search yielded a total of 132 articles, and nine were included after full-text assessment. The selected articles were published between 2004 and 2020 and were grouped as telemedicine systems, algorithms, economic analysis and image quality assessment. Four telemedicine systems that perform a quality assessment, image preprocessing and pathological screening were reviewed. A data and post-deployment bias assessment are not performed in any of the algorithms, and none of the studies evaluate the social impact implementations. There is a lack of representativeness in the reviewed articles, with most authors and target populations from high-income countries and no low-income country representation.

**Conclusions:**

Telemedicine and AI hold great promise for augmenting decision-making in medical care, expanding patient access and enhancing cost-effectiveness. Economic studies and social science analysis are crucial to support the implementation of AI in teleophthalmology screening programs. Promoting fairness and generalizability in automated systems combined with telemedicine screening programs is not straightforward. Improving data representativeness, reducing biases and promoting equity in deployment and post-deployment studies are all critical steps in model development.

## Background

The World Health Organization defines telehealth and telemedicine as the use of information and communication technologies by healthcare professionals to provide diagnosis, treatment and prevent diseases and injuries to patients [1]. Telehealth has a clinical support premise that overcomes geographic distances through information and communication technology for the trade of health guidance [2].

The first telemedicine initiative took place more than one century ago, when telephone use started [3]. After 1970, the transmission of videos, images and audio became more affordable, and in the past three decades, improvements in internet band switch and widespread usage of portable electronics, digital communication devices and wearables contributed to growing interest in telemedicine [3,4].

The COVID-19 pandemic led to restrictions in medical care, ushering in the adoption of digital healthcare solutions to overcome social distancing, including teleconsultation [4,5].

The premise of digital consultations is to reduce distances and represent an alternative to in-person consultation, which is valuable for providing healthcare to remote areas, patients with chronic conditions that need regular care, and patients with mobility restrictions [6].

In ophthalmology, the use of ancillary imaging exams poses a great opportunity for telemedicine and artificial intelligence (AI) algorithm development, with diabetic retinopathy (DR) as the most explored disease. DR is a chronic microvascular ophthalmological complication of diabetes mellitus and remains a leading cause of irreversible blindness among working-aged adults globally [7–9]. The International Council of Ophthalmology recognizes that early diagnosis and treatment are essential to better visual outcomes and recommends a minimum annual dilated ophthalmological exam for every diabetic patient, with the retinal assessment possible through fundus photography [8,10].

Algorithms for DR screening are the most studied and explored field. These models have demonstrated good performance in achieving high sensitivity and specificity, particularly in the detection of even mild cases and vision-threatening diabetic patients [11–14].

The benefits of teleretinal screening for DR programs for DR have been established and recognized worldwide, with standards and guidelines published by the American Telemedicine Association [15]. While telemedicine can reduce geographic restrictions, AI systems have the potential to address the limited healthcare providers’ availability, improve workflow and enhance decision-making. Nevertheless, fair and generalizable algorithms and equitable outcomes are challenging for healthcare AI systems implementation.

This article aims to compare AI systems applied in DR telemedicine screening studies, currently deployed systems, fairness initiatives and the challenges for implementation.

## Methods

This review includes AI-enabled telemedicine programs focused on DR screening, comparing the article’s authors, model technical parameters, metrics results and limitations. For the author’s affiliation and the article’s population classification, we considered the World Bank Country and Lending Groups classification in the publication year as high-income, upper-middle, lower-middle (LMIC) and low-income countries (LIC) [16].

### Literature search

The literature search included PubMed, EMBASE and MEDLINE databases. The search strategy used was the combination of key terms:

(‘telemedicine’ [MeSH] OR ‘remote consultation’ [MeSH] OR ‘teleconsultation’ [tiab]) AND (‘diabetic retinopathy’ [MeSH] OR ‘Diabetes mellitus’ [Mesh]) AND (‘machine learning’ [MeSH] OR ‘machine learning’ [tiab] OR ‘Artificial Intelligence’ [tiab] OR ‘artificially intelligent’ [tiab] OR ‘Artificial Intelligence’ [MeSH] OR ‘Algorithms’[MeSH] OR ‘algorithm*’ [tiab] OR ‘deep learning’ [tiab] OR ‘neural network*’ [tiab] OR ‘neural networks, computer’ [MeSH] OR ‘intelligent machine*’ [tiab]), on 1 September 2022.

Two authors (LFN and LZR) performed the article evaluation process. The first screening excluded non-human studies, articles with a language different from English, Portuguese and Spanish, and non-telemedicine and DR articles according to title analysis. The second screening excluded non-telemedicine and DR articles after the abstract review. The articles were grouped as computational models, image quality, economic analysis and teleophthalmology screening descriptions.

## Results

**Figure 1.**
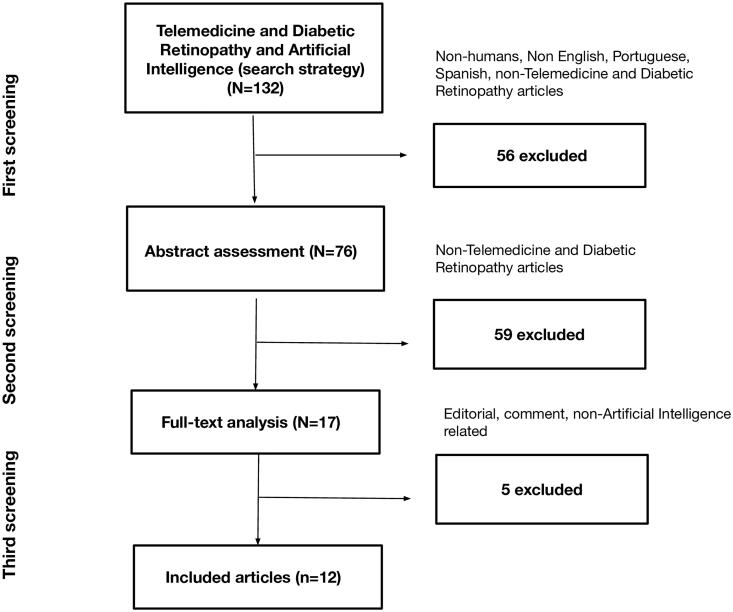
Articles assessment flowchart.

The search strategy identified a total of 132 articles. In the first screening, 56 articles were excluded (non-humans, language screening, non-telemedicine and DR), and in the second screening, 59 articles were excluded (abstract review). The remaining 17 articles were eligible for full-text analysis. One editorial, one comment, three review articles and three unrelated to DR and AI articles were excluded after a full-text analysis (Figure 1).

### General analysis

Our review included nine articles published between 2004 and 2020. The articles include telemedicine systems models, algorithms, image quality models and economic analysis.

The author’s affiliations were from 10 different countries, with the United States (26.67%) and the United Kingdom (13.33%) having the highest representation. We found that 86.67% of the authors were from high-income countries, 6.67% from upper-middle-income countries and 6.67% from lower-middle-income countries. Among the corresponding authors, the majority, specifically 88.89%, hail from high-income nations, while the remaining 11.11% represent lower-middle-income countries. Notably, in four articles, collaborative efforts extended across countries, yet these collaborations exclusively featured authors from high-income countries.

**Figure 2.**
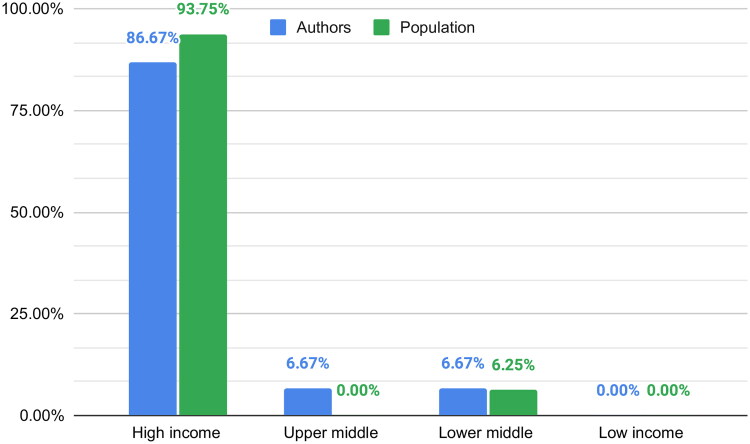
Article’s authors and populations.

The article’s populations were from nine different countries, with the United States being the most represented (25%). We found that 93.75% of the population was from high-income countries, while the remaining 6.25% was from LMIC. None of the studies were conducted in LIC (Figure 2).

### Telemedicine systems

Five articles discuss telemedicine systems to assist the DR screening process, published from 2004 to 2019. Two articles from Hejlesen et al. and Schneider et al. from 2004 and 2005, respectively, presented an internet-based digital communication platform called TOSCA. The platform enables data transfer and database construction across England, Germany, Ireland and Denmark [17,18]. The image analysis routine initiates with image polynomial transformation to enable alignment according to blood vessels, followed by preprocessing and retinal lesions extractions. Next, the image underwent a classification step using supervised algorithms: Bayes Classifier, Mahalanobis distance classifier and K Nearest Neighbor classifier. Lastly, the platform aims to construct a normative reference database to evaluate algorithms and further research [17]. However, no information about the data included for algorithm development or bias control is available.

The article from Joshi and Sivaswamy, in 2011, proposed a web-based DR telescreening framework called DrishtiCare [19]. The proposed platform receives image and clinical information from an external collecting site and performs real-time image quality assessment before data transfer. The server-based prescreening model selects abnormal exams and refers them for specialist evaluation. The images were preprocessed, and the retinal abnormalities were detected and highlighted [19]. The DrishtiCare platform applies automated quality assessment, image screening and lesion detection. The system was evaluated in three primary eye Indian hospitals and is currently not deployed in clinical practice. Data and algorithm details and bias assessment are not reported in the platform [19].

The article from Karnowski et al., in 2013, proposed the Telemedical Retinal Image Analysis and Diagnosis (TRIAD) network for retinal image analysis [20]. The system included an automated quality assessment, vascular, optic nerve, macular structures detection and lesion identification. The quality assessment was done using a support vector machine algorithm, which assigned a quality ranking based on vascular segmentation and measurements in localized neighbourhoods [20]. The optic disc and macula detection establish a coordinate system and remove false positives using the local linearity of the vessels, piecewise connectivity and vessel brightness with a Gaussian-like profile [21]. The abnormality detection consisted of custom detectors to identify the most prominent lesions, such as microaneurysms, exudates, drusen and other findings [20]. In the TRIAD network, the authors did not describe the dataset used or bias control measures.

The article from Saeed et al., in 2019, evaluated a telemedicine system to detect pathological DR changes in retinal fundus photos [22]. The group proposed a cloud-based ophthalmic system applied to a Polish population. The system included image preprocessing by converting the image to grayscale with green channel, histogram stretching, medial filtering and gamma correction. The following step consisted of vascular pattern extraction using vessel segmentation and binarization, with vascular and optic disc removal. Lastly, the pathological changes were identified in the image classification step, and the image was classified as healthy if no findings were present. The reported results are from a 100 images dataset with 98% accuracy, 100% sensitivity and 96% specificity in detecting pathological images. The proposed system showed high sensitivity, specificity and accuracy in the validation set; however, no details of the algorithm architecture, included dataset, and bias control are reported [22].

### Algorithms

Two articles discuss automated algorithms applied for DR screening. The article from Ogunyem et al., in 2014, described a model to predict patients at a high risk of developing DR using clinical variables from 513 patients with type 2 diabetes in South Los Angeles, California. The group applied a Bayesian network and radial basis neural network in Weka [23] and reported model metrics of 28.5% sensitivity, 93% specificity and an AUC of 0.69 using all variables. The model including the six most important features reported a metric of 26.2% sensitivity, 94.5% specificity and 0.71 AUC [23]. The article does not report bias control and is not designed as a teleophthalmological system.

The article from Walton et al. evaluated the effectiveness of the Intelligent Retinal Imaging System (IRIS) in detecting vision-threatening DR and compared its performance to a reading centre interpretation [24]. The IRIS was developed in 15,015 patients from the Harris Health System, Texas. The reported sensitivity was 66.4%, with a false-negative rate of 2% and a specificity of 72.8% compared to the reading centre interpretation [24]. The IRIS is a proprietary system, and details of model development, applied dataset and bias assessment are not provided.

### Economic analysis

One article discusses the economic analysis of AI deployment in DR telescreening. The article from Xie et al. compared the outcome of two deep learning systems: a semi-automated model used as abnormal triage, a fully automated system, and the current human DR assessment. The applied Deep Learning System is an ensemble model of three neural networks (VGGNet, ResNet and DenseNet) trained using 76,370 Singaporean images to detect referable DR. The study was carried out and integrated into the Singaporean Diabetic Retinopathy Screening Program [25].

In the study, the semi-automated model screening model was the least expensive, while the human assessment was the most expensive. There was no statistical difference in referred DR patients across the groups, and the analysis was carried out until the presence of DR was detected or excluded. The study estimates a 20% cost savings in DR screening by switching from the current human assessment ($77 per patient per 12 months) to a semi-automated assessment ($62 per 12-month total) and 14.3% by switching to a fully automated assessment ($66 per patient in 12 months) [25].

In the sensitivity analysis, the cost of human graders, screening specificities and information technology costs were the most influential variables. The automated model specificity was the most important factor affecting the cost difference. The lack of generalizability of the findings is a reported limitation, with further research needed to address labour costs, model performance and information technology infrastructure barriers in LMIC.

### Quality assessment

Quality assessment is one of the steps of ophthalmological telemedical systems. The article from Saha et al., in 2018, evaluates quality assessment methods applied in the triage of retinal fundus photos. The first step consisted of image pre-processing, including pixels cropping and mask application. For the quality screening, the authors applied an AlexNet neural network model trained and evaluated on 7000 images from the EyePACS dataset relabelled by an ophthalmologist based on acceptance criteria. However, the unbalanced nature of the dataset limits the report of the results, and the article does not provide a bias assessment [11].

## Discussion

DR is the leading cause of blindness in work-age adults and the main target for teleretinal screening programs. In our review, we found that although telemedicine and digital healthcare are increasingly implemented even in LMIC, there are still few studies that evaluate the integration and deployment of automated systems into DR telescreen programs [26].

The FDA has approved some AI-assisted DR systems that are currently in clinical use, including IDx-DR, EyeArt and AEYE, which perform quality assessments and detect referable DR for specialist evaluation (Table 1). Additionally, they can be integrated with a telemedical system [12–14]. Among the reviewed systems, none is currently deployed in clinical practice, and there is a lack of bias assessment in data inclusion, preprocessing and modelling steps. The included devices have variable reported sensitivity and specificity for DR screening, which in turn gives rise to concern regarding their reliability and accuracy when deployed in real-world clinical settings.

**Table 1.** FDA approved diabetic retinopathy screening devices.

	IDx-DR v2.3	Eynuk Eyeart v.2.1.0	AEye
Outcome	More than mild diabetic retinopathy in adults	More than mild diabetic and vision-threatening retinopathy	More than mild diabetic retinopathy in adults
Inputs	Macula and disc-centred images	Macula and disc-centred images	Macula and disc-centred images or macula centred
Camera	Topcon NW400	Canon CR-2AF, Canon CR-2 Plus AF	Topcon NW400
Pivotal study Sensitivity	82.24%	94.9–100% more than mild retinopathy, 88.9–100% vision threatening	93% with 1 image, 94.7% with 2 images
Pivotal study Specificity	85.47%	86.7–92% more than mild retinopathy, 93.8–97.5% vision threatening	91.4% with 1 image, 88.6% with 2 images
Algorithms datasets	Not described	Not described	Not described
Bias assessment	Using detectors designed to detect racially invariant biomarkers to minimize the risk of ethnic or racial bias in algorithm output, and spectrum bias assessment	Not described	Not described

Biases in AI models are a growing concern in clinical decision software, with unfair predictions made in new data and minorities resulting from unbalanced datasets and non-satisfactory monitoring and re-calibration processes. Examples of biased algorithms and inherited data biases are algorithms that miss sepsis diagnosis in underrepresented populations [27,28], underdiagnose melanoma in dark-skin tones [29] and language models that show gender bias [30].

There is a lack of representativeness in the reviewed articles, with no authors coming from LIC, and the majority coming from high-income countries. There are also no studies that evaluate LIC as the target population, with no representation from South America, Africa and Oceania continents. In LMIC countries, the burden of a lack of healthcare professionals and medical specialists is higher, and AI has the potential to overcome this problem; however, more representativeness is needed.

There is a no social science investigation that appraises the impact of telemedical AI-assisted programs among the reviewed articles. Social science studies help to understand the unintended social consequences of AI implementation in each healthcare system.

Although many studies evaluate the cost-effectiveness of telemedicine programs, the study from Xie et al. is the first that evaluates the economic consequences of implementing an automatic screening process into teleophthalmological screening programs [25]. The study concluded that semi-supervised models are more cost-effective for DR screening in the Singaporean scenario, with graders’ cost, screening specificities and IT costs as the most influential variables. Additional studies addressing different healthcare and economic scenarios are needed to evaluate the generalizability of AI implementation in DR screening telehealth. For a cost-effectiveness analysis, the screening uptake rates, grader and information technology labour costs, and model specificity need to be considered according to the healthcare system.

### Challenges for AI-assisted teleophthalmological screening

#### Training and workflow integration

Ophthalmological imaging exams require operator training and basic anatomy and ocular diseases knowledge for good performance. AI-enabled devices, however, require further additional software use training and how to interpret the results and make decisions. The deployment of algorithms must integrate the existing workflow, provide reliable performance and a positive healthcare experience. However, challenges can arise when implementing AI systems, such as Google research DR screening algorithm, which demonstrated good performance but failed to integrate into the existing image collection workflow. This led to delays, frustration among healthcare professionals, and unnecessary returns [31,32]. Constantly training and educating healthcare professionals are also necessary to ensure that they are equipped to work with these complex digital systems.

#### Economic and infrastructure

Although equipment prices are a possible limiting factor, retinal camera costs become less important for large population-based screening programs. The implementation of AI systems in screening programs requires additional infrastructure, including internet access, a data transferring platform, and information technology personnel, which can increase exam costs. Internet access can be a limiting factor in remote areas, but new satellite internet access providers and retinal cellphone cameras can potentially expand connectivity.

Beyond the initial deployment, post-deployment monitoring and recalibration demand specialized data and healthcare professionals, which can be an additional barrier to access. Furthermore, differences in healthcare systems and countries’ economic circumstances can hinder the widespread deployment of AI-assisted DR screening programs.

Cost-effectiveness is measured according to a country’s annual gross domestic product per capita and can differ according to the healthcare systems. The incremental cost-effectiveness ratio, which measures the difference in cost between intervention and treatment effect, supports the cost-effectiveness of AI-assisted DR screening; however, few studies evaluate integrating AI systems in telemedicine screening programs [33–35]. Furthermore, the absence of systematic national DR screening initiatives in several countries, such as the United States, serves as a hindrance to fully harnessing the advantages of integrating AI technology into telemedicine screening programs.

#### Biases

Biases are a growing concern in the development and deployment of AI systems, with numerous reports of problematic deployments in sepsis diagnosis, COVID and patient triage [27,36,37]. One major source of bias comes from the use of nonrepresentative data in algorithm training, which can result in biased models against underrepresented populations.

In ophthalmology, the majority of datasets are from developed countries, which limits the generalizability of AI algorithms to other regions [38]. To address this problem is crucial to ensure that data used in AI model development are representative of the target population. Comprehensive data analysis during the system development and monitoring and recalibration after deployment are fundamental to ensure fairness and accuracy in real-world settings.

The assessment of adequate results metrics, bias and social impacts in post-deployed AI systems is yet an unsolved problem in healthcare. Adequate metrics and assessment tools are needed to evaluate the impact of AI deployments and ensure that AI does not perpetuate or amplify existing biases in the healthcare systems.

#### Health equity

The telemedicine premise of digitally exchanging information can be problematic in patients who lack access or are not familiar with technology, as well as in countries with inadequate telemedicine infrastructure. This can create barriers to healthcare, particularly for underrepresented populations and social groups. While telemedicine initiatives have been shown to be particularly beneficial in LMIC, where geographic distances and shortage of medical professionals can be challenges, the deployment of AI models in these countries can exacerbate existing disparities and be dangerous to those populations.

It is crucial to carefully evaluate the potential risks and benefits of deploying AI systems in healthcare and to make efforts to mitigate the negative impacts on underrepresented populations. This includes addressing the underlying social determinants of health, as well as ongoing monitoring and recalibration of AI models to ensure that they are accurate and equitable. By prioritizing fairness and accessibility in the development and deployment of AI systems, we can help to ensure that telemedicine and other technological solutions fulfil their potential to improve healthcare outcomes for all.

## Conclusions

Telemedicine and AI hold great promise for augmenting decision-making in medical care, expanding patient access and enhancing cost-effectiveness. DR screening has been one of the leading applications of AI in ophthalmology, with three US FDA-approved programs that are deployed in the clinical setting. The use of AI-enabled systems has the potential to streamline and optimize the screening process. In our review, the articles proposed AI systems for telemedicine, including quality assessment, preprocessing and pathological classification.

AI systems can reflect the existing biases in healthcare, and promoting fairness and generalizability in automated systems is not straightforward. Improving data representativeness, reducing biases and promoting equity in deployment and post-deployment studies are all critical steps in achieving more equitable outcomes.

The lack of deployed telemedicine AI-assisted programs provides an opportunity to prioritize fair AI algorithms that promote health equity. Comprehensive post-deployment studies that assess not only model biases but also impact and recalibration are needed to ensure that these technologies are deployed in a way that promotes fairness and improves outcomes for every patient.

## Data Availability

The included data and analysis are available under request.
